# Exercise-based interventions for postoperative rehabilitation in breast cancer patients: A systematic review and meta-analysis of randomized controlled trials

**DOI:** 10.1097/MD.0000000000043705

**Published:** 2025-08-22

**Authors:** Tingyu Xue, Li Zhang, Dairong Zhang

**Affiliations:** a China Three Gorges University, Hubei, China; b AnQing Municipal Hospital, Auhui, China; c Yichang Second People’s Hospital, Hubei, China.

**Keywords:** breast cancer, exercise intervention, lymphedema, meta-analysis, muscle strength recovery, pain management, postoperative rehabilitation, quality of life, randomized controlled trial, shoulder joint function

## Abstract

**Background:**

This systematic review and meta-analysis aim to evaluate the effects of exercise interventions on pain, lymphoedema, shoulder joint range of motion (ROM), muscle strength, and quality of life in postoperative breast cancer patients, and to provide evidence-based recommendations for clinical practice.

**Methods:**

This systematic review and meta-analysis adhered to PRISMA guidelines and was registered on PROSPERO (CRD420251045309). A thorough search was performed in PubMed, Web of Science, Cochrane Library, and Embase for randomized controlled trials evaluating the effect of exercise on postoperative recovery in breast cancer patients. Data on pain, lymphoedema, ROM, muscle strength, and quality of life were extracted and analyzed using RevMan 5.4 software. The results were synthesized using weighted mean differences and odds ratios.

**Results:**

A total of 22 randomized controlled trials with 2305 patients were included in the meta-analysis. Exercise interventions significantly reduced postoperative pain (mean difference = −0.49, 95% confidence interval: −0.71 to −0.27, *P* < .0001) and improved muscle strength across various muscle groups. Exercise was also effective in reducing the incidence of lymphoedema (odds ratio = 0.34, 95% confidence interval: 0.19–0.61, *P* = .0003) and improving shoulder ROM, particularly in flexion, extension, abduction, and adduction. In terms of quality of life, exercise enhanced physical function, role function, and emotional well-being, and reduced fatigue and appetite loss.

**Conclusion:**

Exercise interventions are beneficial for improving pain management, lymphoedema control, upper limb function, muscle strength, and overall quality of life in postoperative breast cancer patients. These findings support the inclusion of exercise as a key component of postoperative rehabilitation. Future research should focus on optimizing exercise protocols and exploring long-term effects on breast cancer survivors.

## 1. Introduction

Breast cancer (BC) remains one of the most prevalent malignancies among women worldwide.^[1]^ Although advances in screening technologies and therapeutic strategies have led to continuous improvements in patient survival rates, numerous complications associated with BC treatments continue to exert a profound impact on postoperative quality of life (QoL).^[2]^ Common postoperative complications, including upper limb dysfunction, pain, lymphedema, muscle weakness, and emotional distress, are particularly pronounced during the early stages of rehabilitation and may persist over time, significantly limiting patients’ ability to perform daily activities and participate in social functions.^[3–5]^

In recent years, exercise has garnered increasing attention as a crucial intervention in postoperative rehabilitation for BC patients.^[6]^ A growing body of research has demonstrated the multifaceted benefits of exercise in facilitating postoperative recovery.^[7]^ Firstly, exercise contributes to the alleviation of postoperative pain. By promoting blood circulation, improving muscle condition, and enhancing joint flexibility, exercise helps reduce pain symptoms in the shoulder and upper limbs, thereby decreasing the incidence of chronic pain.^[8]^ Secondly, in terms of lymphedema prevention and management, regular exercise enhances venous and lymphatic return, reducing the risk of limb swelling and mitigating the severity of existing lymphedema symptoms.^[9]^ Regarding upper limb functional recovery, exercise improves the range of motion (ROM) of the shoulder joint and strengthens the muscles of the shoulder and arms, thereby enhancing mobility and facilitating the restoration of daily functional abilities. Exercise interventions also significantly boost muscle strength, increase the upper limbs’ load-bearing and endurance capacities, and help lower the risk of postoperative frailty and disability.^[10]^ Functional assessment tools, such as the disabilities of the arm, shoulder and hand (DASH) questionnaire, have shown that patients who engage in exercise interventions exhibit markedly better upper limb functional scores compared to those who do not.^[11]^ Furthermore, exercise plays a pivotal role in improving overall QoL. Studies utilizing BC-specific QoL instruments (e.g., FACT-B) as well as general assessment tools (e.g., QLQ-C30) have confirmed that exercise interventions enhance multiple dimensions of QoL, including physical, emotional, social, and role functioning.^[12]^

Importantly, regardless of exercise type – whether aerobic, resistance, stretching, or balance training – when appropriately prescribed in terms of frequency, intensity, and duration, exercise interventions are generally safe, feasible, and effective for rehabilitation purposes. Current clinical guidelines broadly recommend that BC survivors engage in individualized and structured exercise programs under professional supervision to promote comprehensive recovery and improve long-term outcomes.

However, despite the extensive clinical research exploring the effects of exercise interventions on postoperative recovery, considerable heterogeneity exists across studies in terms of intervention types, program designs, and outcome measures. The existing evidence has not yet been systematically integrated. Therefore, the present study aims to conduct a systematic review and meta-analysis to comprehensively evaluate the effects of exercise interventions on major outcomes in postoperative BC patients, including pain, lymphedema, shoulder joint function, muscle strength, and QoL. This review seeks to clarify the value and limitations of exercise in postoperative rehabilitation, provide evidence-based guidance for clinical practice, and offer directions for future research.

## 2. Methods

### 2.1. Study design

This systematic review and meta-analysis was performed in full compliance with the preferred reporting items for systematic reviews and meta-analyses statement (Table S1, Supplemental Digital Content, https://links.lww.com/MD/P696). The study protocol was prospectively registered in the international prospective register of systematic reviews under the registration ID CRD420251045309.

### 2.2. Literature search and selection

Two researchers independently developed a search strategy focusing on exercise interventions and postoperative rehabilitation in BC patients. The following keywords and their combinations were used: (“exercise” OR “physical activity” OR “training” OR “sports” OR “rehabilitation”) AND (“breast cancer” OR “breast neoplasms”) AND (“postoperative” OR “after surgery” OR “surgical recovery”). We systematically searched 4 major databases – PubMed, Web of Science, Cochrane Library, and Embase – for relevant publications up to May 2025. To enhance the comprehensiveness of the search, we additionally examined the bibliographies of all included articles to identify further studies that met the eligibility criteria but were not retrieved through the primary database queries.

### 2.3. Inclusion and exclusion criteria

Study selection was based on the PICOS framework: (1) population: patients who had undergone surgical treatment for BC; (2) intervention: any form of exercise intervention; (3) comparison: routine postoperative rehabilitation management without exercise intervention; (4) outcomes: at least one postoperative rehabilitation-related outcome was assessed, including pain, lymphedema, shoulder ROM, muscle strength, DASH score, FACT-B score, or QLQ-C30 score; and (5) study design: only randomized controlled trials (RCTs) were included. Exclusion criteria were: (1) studies from which required outcome data could not be extracted; (2) abstracts, case reports, animal experiments, or review articles; and (3) duplicate reports or studies with overlapping patient populations, in which case the version with the largest sample size or most complete data was included.

### 2.4. Data extraction

Two researchers independently extracted data using a standardized excel sheet. Extracted information included: 1st author, year of publication, country or region of study, sample size, baseline patient characteristics (such as age), intervention type and duration, control measures, follow-up duration, and outcomes (pain scores, incidence or volume change of lymphedema, ROM, muscle strength, DASH scores, FACT-B scores, and QLQ-C30 scores). Disagreements during data extraction were resolved through discussion or consultation with a 3rd researcher.

### 2.5. Quality assessment

The quality of the included RCTs was assessed using the Cochrane Collaboration’s risk of bias 2 tool. This assessment covered essential aspects of study methodology, such as random sequence generation, allocation concealment, blinding procedures, management of incomplete outcome data, and the risk of selective outcome reporting. Based on these evaluations, studies were classified as having a low, high, or unclear risk of bias.

### 2.6. Assessment of publication bias

Publication bias was assessed using funnel plots. If significant bias was detected, sensitivity analyses were conducted to further explore its potential impact.

### 2.7. Statistical analysis

All meta-analyses were performed using RevMan 5.4 software (Cochrane Collaboration, Oxford, UK). For continuous variables, such as pain scores, ROM, muscle strength, and QoL measures (DASH, FACT-B, and QLQ-C30), either mean differences (MDs) or standardized mean differences were calculated, accompanied by 95% confidence intervals (CIs). For categorical outcomes like the incidence of lymphedema, odds ratios (ORs) with 95% CIs were used. In cases where only medians and interquartile ranges were provided, conversion to means and standard deviations was carried out based on methods from Luo et al and Wan et al.^[13,14]^ Heterogeneity between studies was assessed using the chi-squared (Q) test and quantified with the *I*^2^ statistic. A random-effects model was applied when significant heterogeneity was detected (*I*^2^ > 50% or *P* < .05); otherwise, a fixed-effects model was used. All statistical tests were two-tailed, with significance set at a *P* value <.05.

## 3. Results

### 3.1. Study selection and baseline characteristics

**Figure 1.**
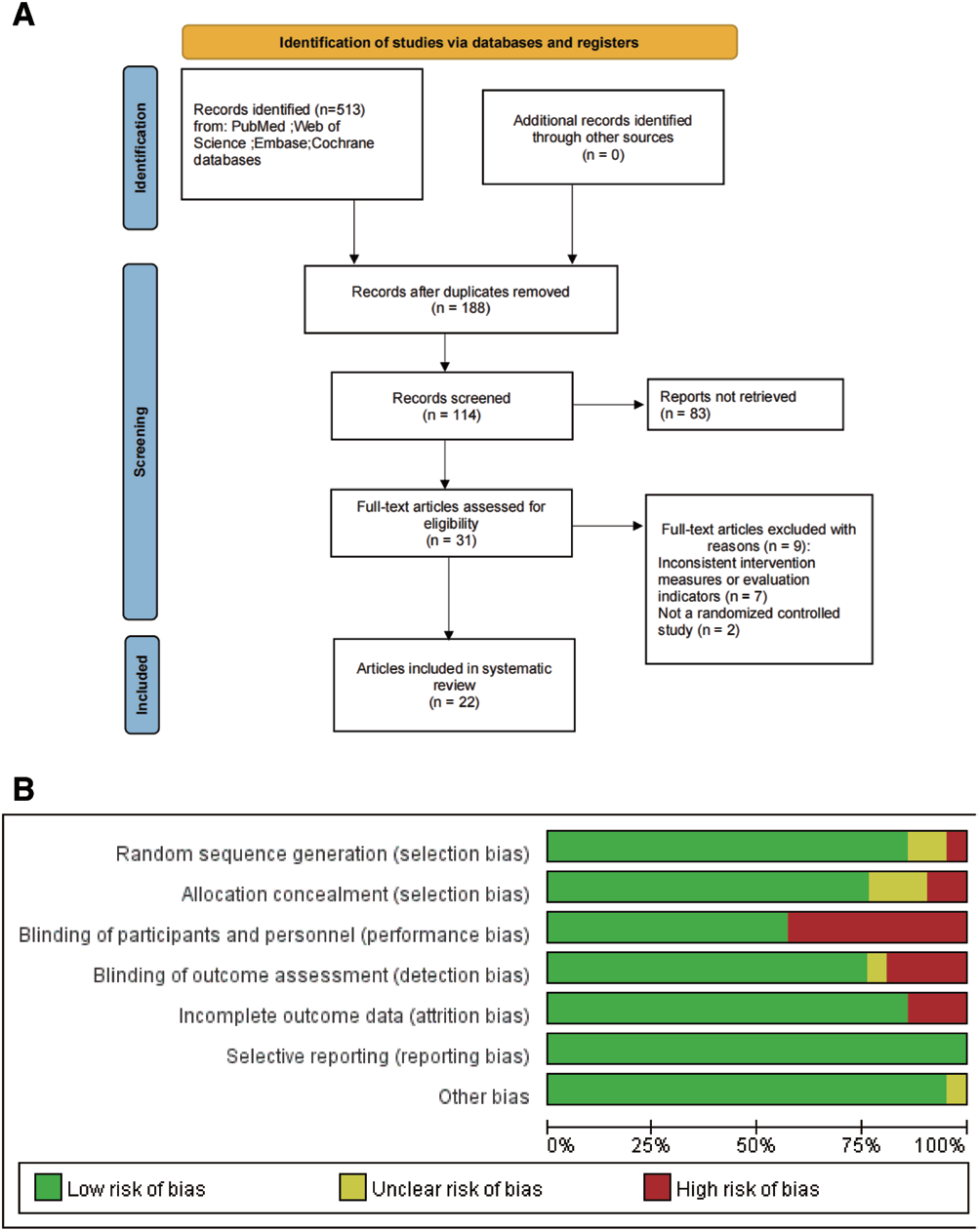
(A) PRISMA flowchart; (B) quality assessment of included studies. PRISMA = preferred reporting items for systematic reviews and meta-analyses.

The preliminary search identified 513 relevant references. After removing duplicates and undergoing detailed screening, 22 articles were deemed eligible for the final analysis (Fig. 1A).^[6,12,15–32]^ These studies included a total of 2305 patients, with 1254 in the exercise group and 1051 in the control group. All studies included in this meta-analysis were retrospective case-control designs, with publication dates ranging from 2002 to 2024. A summary of the key study features and patient demographics is provided in Table 1. According to quality assessment, all articles were considered of moderate standard (Fig. 1B).

**Table 1:** Study characteristics.

References	Country	Study type	Sample size (intervention/control)	Age (years; (intervention/control))	Surgery type	Intervention	Time	Evaluation Index
Williams et al^[33]^	UK	RCT	15/16	59.7 (2.1)/59.3 (2.4)	Breast cancer surgery	Lymphatic drainage	3W	II(1), II(3), IV(2)
Todd et al^[32]^	UK	RCT	58/58	56.5 (12.4)/57.2 (14)	Axillary lymph node dissection	Shoulder and elbow exercise	NA	III(2), III(1), III(7), III(9), II(2)
Torres Lacomba et al^[34]^	Spain	RCT	60/60	52.9 (10.7)/52.9 (12.5)	Unilateral breast cancer surgery with axillary lymph node dissection	Lymphatic drainage and Shoulder and elbow exercise	3W	II(1), II(2), II(5)
Hayes et al^[19]^	Australia	RCT	67/60;67/60	51.2 (8.8)/53.9 (7.7); 52.2 (8.6)/53.9 (7.7)	Breast cancer surgery	Resistance training and Aerobic exercise	8M	I(1), III(3), III(1), III(5), IV(1), II(4)
Kilbreath et al^[23]^	Australia	RCT	81/79	53.4 (12.1)/51.6 (11.0)	Sentinel node biopsy/ axillary node dissection	Resistance training and Massage	6M	III(2), III(6), III(7), II(2)
Loudon et al^[25]^	Australia	RCT	15/13	55.1 (2.5)/60.5 (3.6)	Breast cancer surgery	Aerobic exercise	8W	I(2), II(1), II(2), II(4)
Harder et al^[20]^	UK	RCT	46/46	54.6 (10.9)/55.8 (11.6)	Axillary lymph node dissection	Aerobic exercise	10W	I(2), III(3), III(1), III(7), III(4), II(5)
Park^[28]^	Korea	RCT	35/34	54.78 (3.42)/52.48 (5.57)	Breast cancer surgery	Resistance training and Aerobic exercise	4W	I(2), III(2)
Tambour et al^[31]^	Denmark	RCT	38/35	62.0 (11.5)/60.9 (10.8)	Breast cancer surgery	Lymphatic drainage	4W	II(1), II(5)
Pasyar et al^[27]^	Iran	RCT	20/20	51.6 (10.46)/51.8 (11.4)	Breast cancer surgery	Aerobic exercise	8W	II(1), IV(2)
Sweeney et al^[30]^	US	RCT	50/50	52.8 (10.6)/53.6 (10.1)	Sentinel lymph node biopsy	Resistance training and Aerobic exercise	4M	III(2), III(3), III(7)
Kilbreath et al^[24]^	Australia	RCT	41/47	53.7 (10.4)/59.5 (8.0)	Breast cancer surgery	Resistance training and Aerobic exercise	12W	II(4), II(3), III(6)
Majed et al^[26]^	Lebanon	RCT	30/30	NA	Mastectomy	Shoulder and elbow exercise	4W	III(2), III(1)
Ammitzbøll et al^[15]^	Denmark	RCT	82/76	53 (10)/52 (10)	Axillary Lymph Node Dissection	Resistance training	12M	I(2), I(1)
Bruce et al^[16]^	UK	RCT	196/196	58.4 (12.2)/57.8 (12.0)	Breast cancer surgery	Resistance training	12M	I(2), III(3), III(1), IV(3), II(1)
Bloomquist et al^[21]^	Denmark	RCT	46/22	47.4 (9.4)/50 (9.3)	lumpectomy or mastectomy	Aerobic exercise	12M	II(1), II(2), II(4), III(3), III(6)
Bloomquist et al^[35]^	Denmark	RCT	46/22	47.4 (9.4)/50 (9.3)	lumpectomy or mastectomy	Aerobic exercise	12M	III(7), III(8)
Guloglu et al^[17]^	Turkey	RCT	27/24;26/24	46.0 (7.7)/44.2 (7.0); 48.8 (9.8)/44.2 (7.0)	Breast cancer surgery	Resistance training/ Shoulder and elbow exercise	8W	I(2), III(3), III(7), III(8), IV(3)
Huo et al^[18]^	China	RCT	51/61;50/61	Total:50.5 (11.5)	Modified radical mastectomy/breast-conserving surgery or sentinel lymph node biopsy/BCS/axillary lymph node dissection	Shoulder and elbow exercise/ Massage	NA	I(2), III(2), III(7), III(8)
Antunes et al^[36]^	Portugal	RCT	47/46	49.66 (9.43)/51.02 (9.54)	Breast cancer surgery	Resistance training and Aerobic exercise	20W	III(7), III(8), IV(2)
Min et al^[6]^	Korea	RCT	28/28	50.8 (6.8)/49.9 (6.5)	Breast cancer surgery	Resistance training	6M	III(2), III(7), III(1), III(8), II(2)
Simón et al^[29]^	Spain	RCT	32/28	52.6 (8.8)/52.0 (9.4)	Breast cancer surgery	Resistance training	12W	III(3), III(7), III(8)

Evaluation indices are categorized as follows: I. Pain (① Neuropathic pain; ② VAS/NRS); II. Lymphedema (① Volume of arm lymphedema; ② Incidence of arm lymphedema; ③ Dermal thickness; ④ Extracellular fluid measurement; ⑤ Arm circumference); III. Functional status (① FACT-B/QoL-BC/EQ-5L; ② ROM; ③ DASH; ④ Oxford Shoulder Score [OSS]; ⑤ 3-minute step test; ⑥ EORTC BR23; ⑦ Strength [grip, shoulder muscles, 1RM]; ⑧ Other functional metrics [endurance, 6-minute walk, chair stand]; ⑨ Shoulder Disability Questionnaire [SDQ]); IV. Quality of life (① FACIT; ② EORTC QLQ-C30; ③ Other tools [SF-12, EQ5D-5L, GRC]).

RCT = randomized controlled trial.

### 3.2. Pain

**Figure 2.**
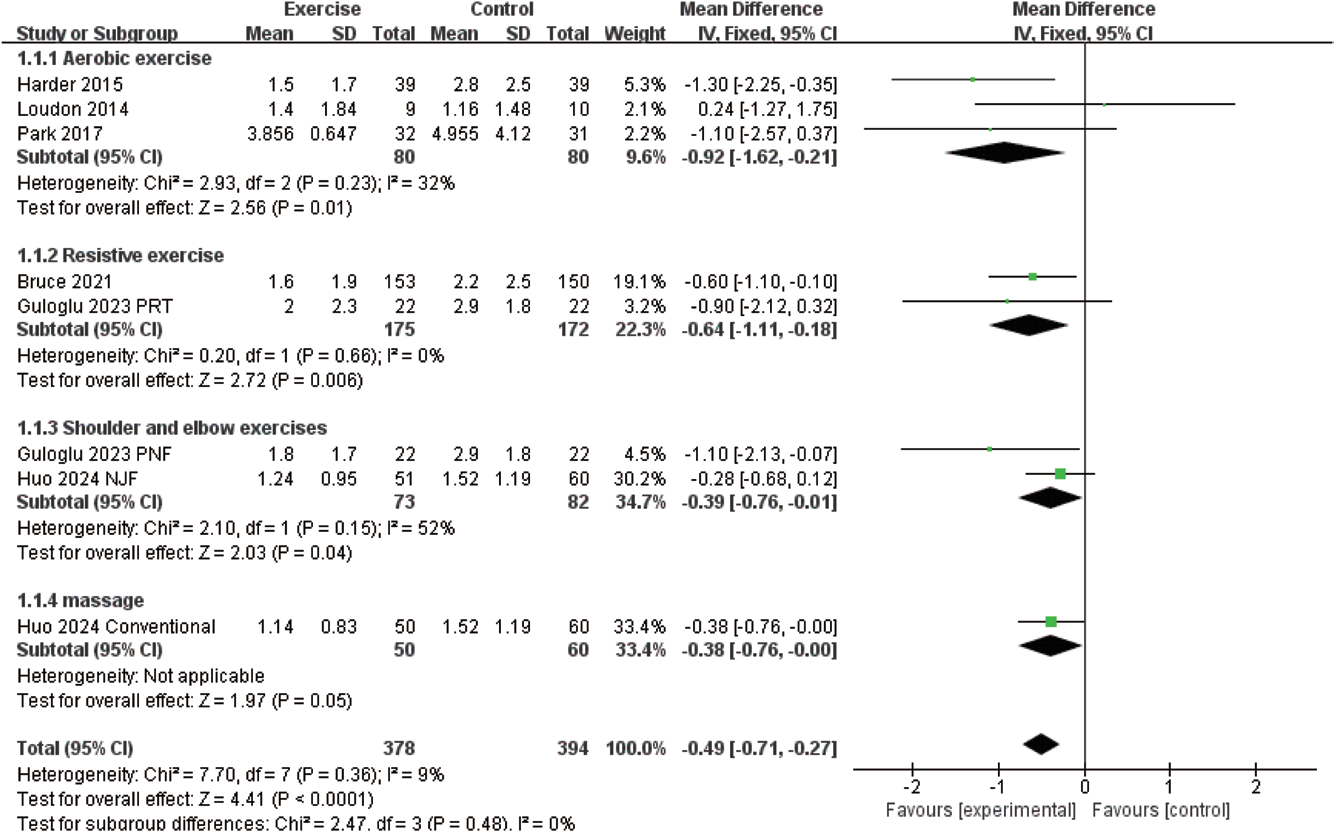
Effect of exercise on postoperative pain.

Exercise interventions were found to significantly alleviate postoperative pain in BC patients. The combined analysis revealed that these interventions substantially reduced pain scores (weighted mean differences [WMD]: −0.49, 95% CI [−0.71, −0.27], *P* < .0001, Fig. 2), with minimal variability between studies (*I*² = 0%). Subgroup analysis confirmed that different types of exercise contributed to pain relief. Aerobic exercise resulted in a marked decrease in pain (WMD: −0.92, 95% CI [−1.62, −0.21], *P* = .01), while resistance training also provided significant pain reduction (WMD: −0.64, 95% CI [−1.11, −0.18], *P* = .006). Exercises focusing on shoulder and elbow mobility were linked to pain reduction (WMD: −0.39, 95% CI [−0.76, −0.01], *P* = .04). Massage therapy showed a slight trend toward pain relief, with results approaching statistical significance (WMD: −0.38, 95% CI [−0.76, 0.00], *P* = .05). Together, these findings highlight the effectiveness of exercise interventions in managing postsurgical pain in BC survivors.

### 3.3. Lymphedema

**Figure 3.**
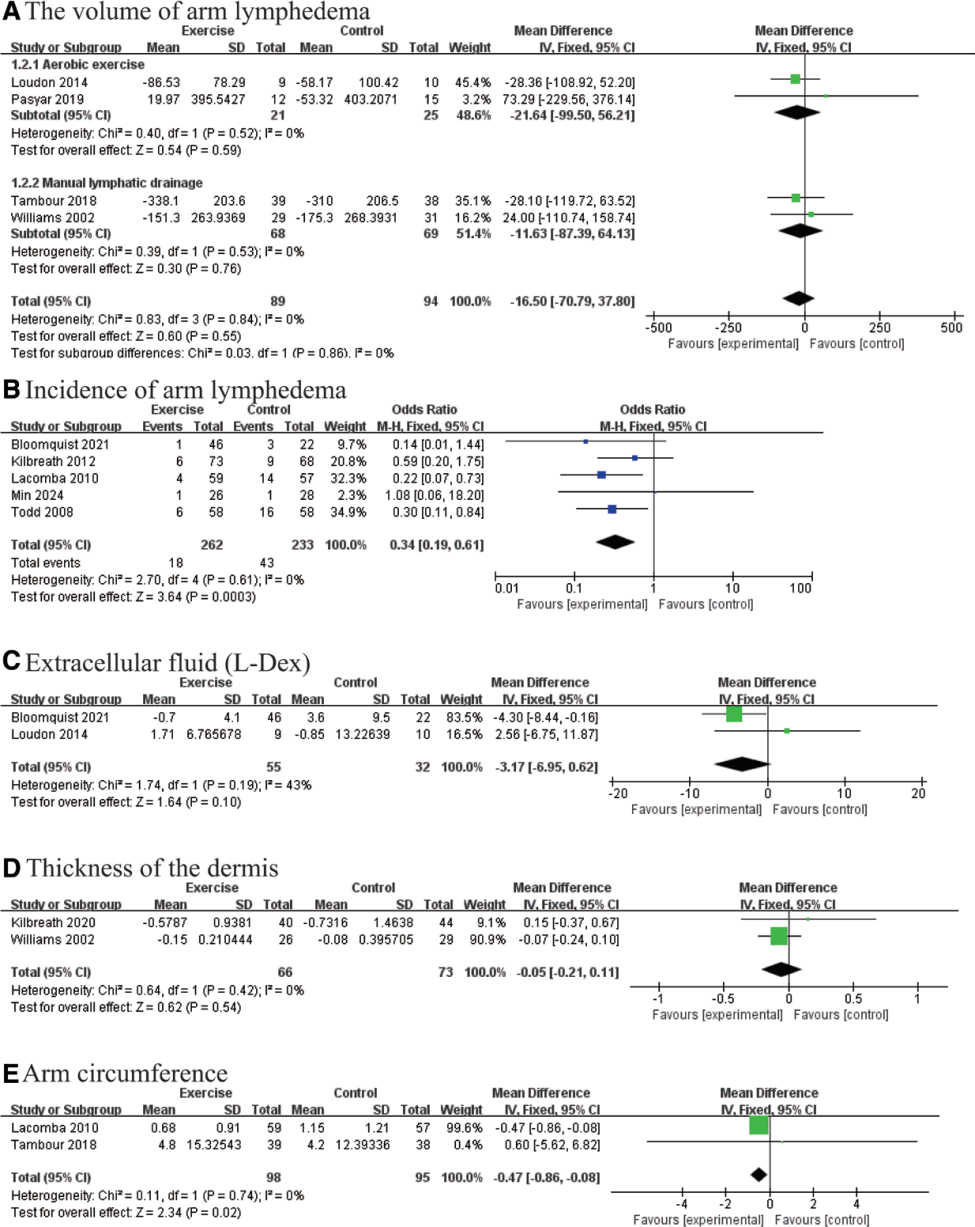
Effect of exercise on lymphedema: (A) effect of exercise on the change in lymphedema volume; (B) effect of exercise on lymphedema incidence; (C) change in extracellular fluid (L-Dex); (D) changes in skin thickness after exercise; and (E) effect of exercise on arm circumference.

Exercise interventions were also found to contribute to the management of lymphedema in postoperative BC patients. Although the difference in lymphedema volume between the intervention and control groups did not reach statistical significance (WMD: −16.50 mL, 95% CI [−70.79, 37.80], *P* = .55; Fig. 3A), the pooled analysis demonstrated a significantly lower incidence of lymphedema in the exercise group (OR: 0.34, 95% CI [0.19, 0.61], *P* = .0003; Fig. 3B). Further analyses revealed a nonsignificant downward trend in extracellular fluid accumulation, as measured by L-Dex values (WMD: −3.17, 95% CI [−6.95, 0.62], *P* = .10; Fig. 3C), as well as a modest reduction in skin thickness (WMD: −0.05 mm, 95% CI [−0.21, 0.11], *P* = .54; Fig. 3D). Notably, arm circumference was significantly decreased in patients receiving exercise interventions compared to controls (WMD: −0.47 cm, 95% CI [−0.86, −0.08], *P* = .02; Fig. 3E), indicating a favorable effect on localized edema. Collectively, while not all indicators reached statistical significance, the overall trend suggests that exercise may play a beneficial role in both reducing the risk and alleviating symptoms of lymphedema.

### 3.4. Range of motion

**Figure 4.**
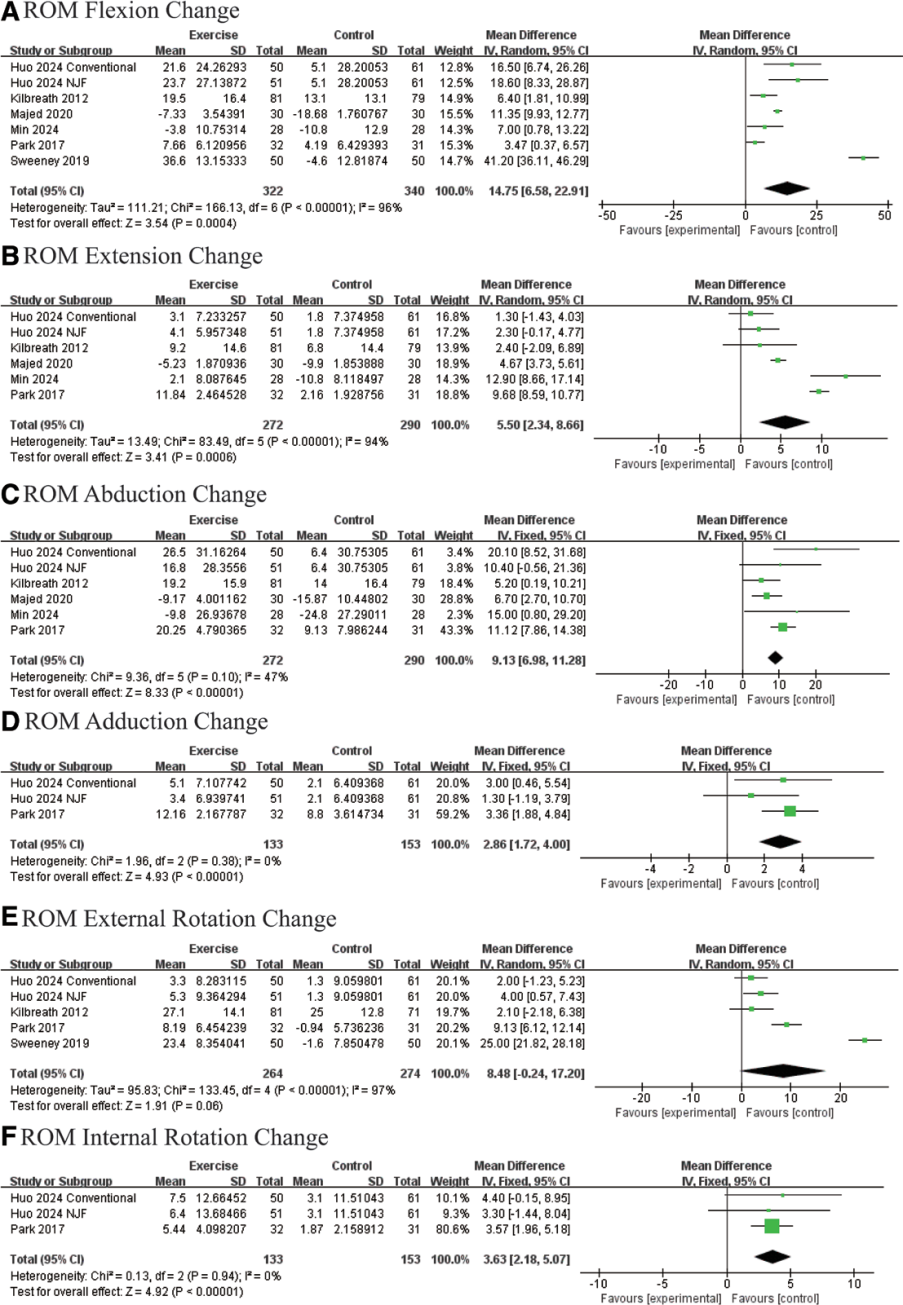
Effect of exercise on range of motion (ROM): (A) flexion; (B) extension; (C) abduction; (D) adduction; (E) external rotation; and (F) internal rotation.

Exercise interventions demonstrated a significant positive impact on the restoration of shoulder ROM following BC surgery. Specifically, notable improvements were observed in shoulder flexion (WMD: 14.75°, 95% CI [12.91, 16.58], *P* = .004; Fig. 4A), extension (WMD: 5.50°, 95% CI [2.34, 8.66], *P* = .0006; Fig. 4B), abduction (WMD: 9.13°, 95% CI [6.98, 11.28], *P* < .00001; Fig. 4C), and adduction (WMD: 2.86°, 95% CI [1.72, 4.00], *P* < .00001; Fig. 4D). Although the enhancement in external rotation did not reach statistical significance (WMD: 8.48°, 95% CI [0.24, 17.20], *P* = .06; Fig. 4E), the trend favored improvement. Internal rotation showed a significant increase in the intervention group (WMD: 3.63°, 95% CI [2.18, 5.07], *P* < .00001; Fig. 4F).

At the conclusion of the intervention period, final ROM assessments continued to favor the exercise group, with significant improvements in flexion (WMD: 15.01°, 95% CI [14.39, 15.62], *P* = .002; Fig. S1A, Supplemental Digital Content, https://links.lww.com/MD/P697), extension (WMD: 6.11°, 95% CI [2.33, 9.90], *P* = .002; Fig. S1B, Supplemental Digital Content, https://links.lww.com/MD/P697), abduction (WMD: 2.32°, 95% CI [1.25, 3.38], *P* < .0001; Fig. S1C, Supplemental Digital Content, https://links.lww.com/MD/P697), and external rotation (WMD: 10.70°, 95% CI [9.29, 12.21], *P* = .05; Fig. S1D, Supplemental Digital Content, https://links.lww.com/MD/P697). No significant difference was observed in internal rotation at the endpoint (WMD: −0.27°, 95% CI [−5.12, 4.58], *P* = .91; Fig. S1E, Supplemental Digital Content, https://links.lww.com/MD/P697). Collectively, these results support the role of exercise in enhancing shoulder mobility across multiple dimensions during postoperative rehabilitation.

### 3.5. Muscle strength

**Figure 5.**
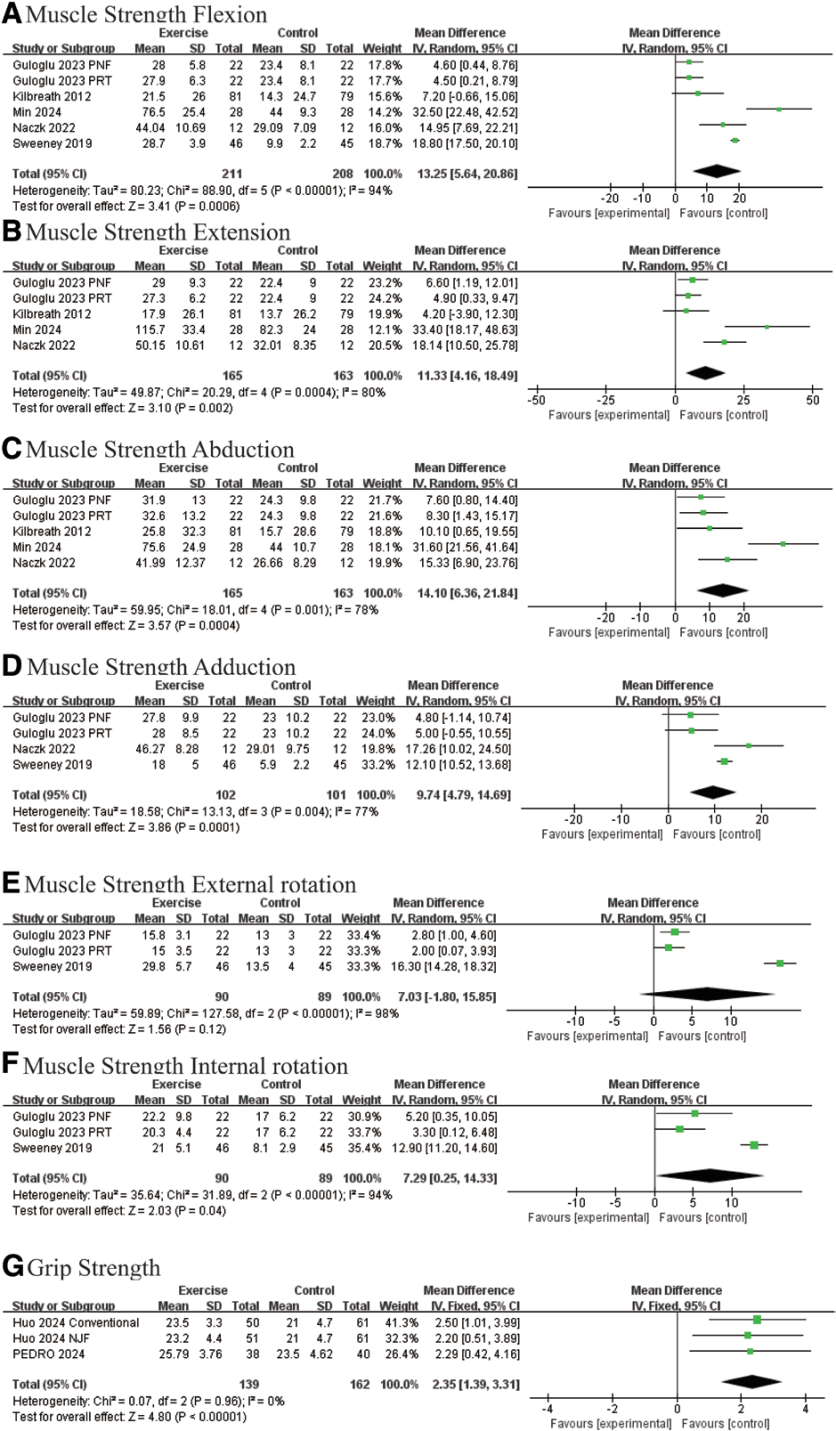
Effect of exercise on muscle strength: (A) flexor strength; (B) extensor strength; (C) adductor strength; (D) internal rotation strength; (E) external rotation strength; (F) internal rotation strength; and (G) grip strength.

Exercise interventions significantly aided muscle strength recovery following BC surgery. Compared to the control group, the exercise group showed notable improvements in flexor strength (WMD: 12.98 kg, 95% CI [4.18, 21.79], *P* = .004; Fig. 5A), extensor strength (WMD: 9.22 kg, 95% CI [1.91, 16.54], *P* = .01; Fig. 5B), abductor strength (WMD: 13.93 kg, 95% CI [4.12, 23.73], *P* = .005; Fig. 5C), and adductor strength (WMD: 7.84 kg, 95% CI [2.12, 13.56], *P* = .007; Fig. 5D). While improvements in external rotator strength did not reach statistical significance (WMD: 7.03 kg, 95% CI [−1.80, 15.85], *P* = .12; Fig. 5E), a positive trend was noted. Furthermore, internal rotator strength showed significant enhancement (WMD: 7.29 kg, 95% CI [0.25, 14.33], *P* = .04; Fig. 5F). Grip strength was also significantly improved (WMD: 2.35 kg, 95% CI [1.39, 3.31], *P* < .00001; Fig. 5G), further highlighting the importance of exercise in muscle strength recovery post-surgery.

### 3.6. QLQ-C30

**Figure 6.**
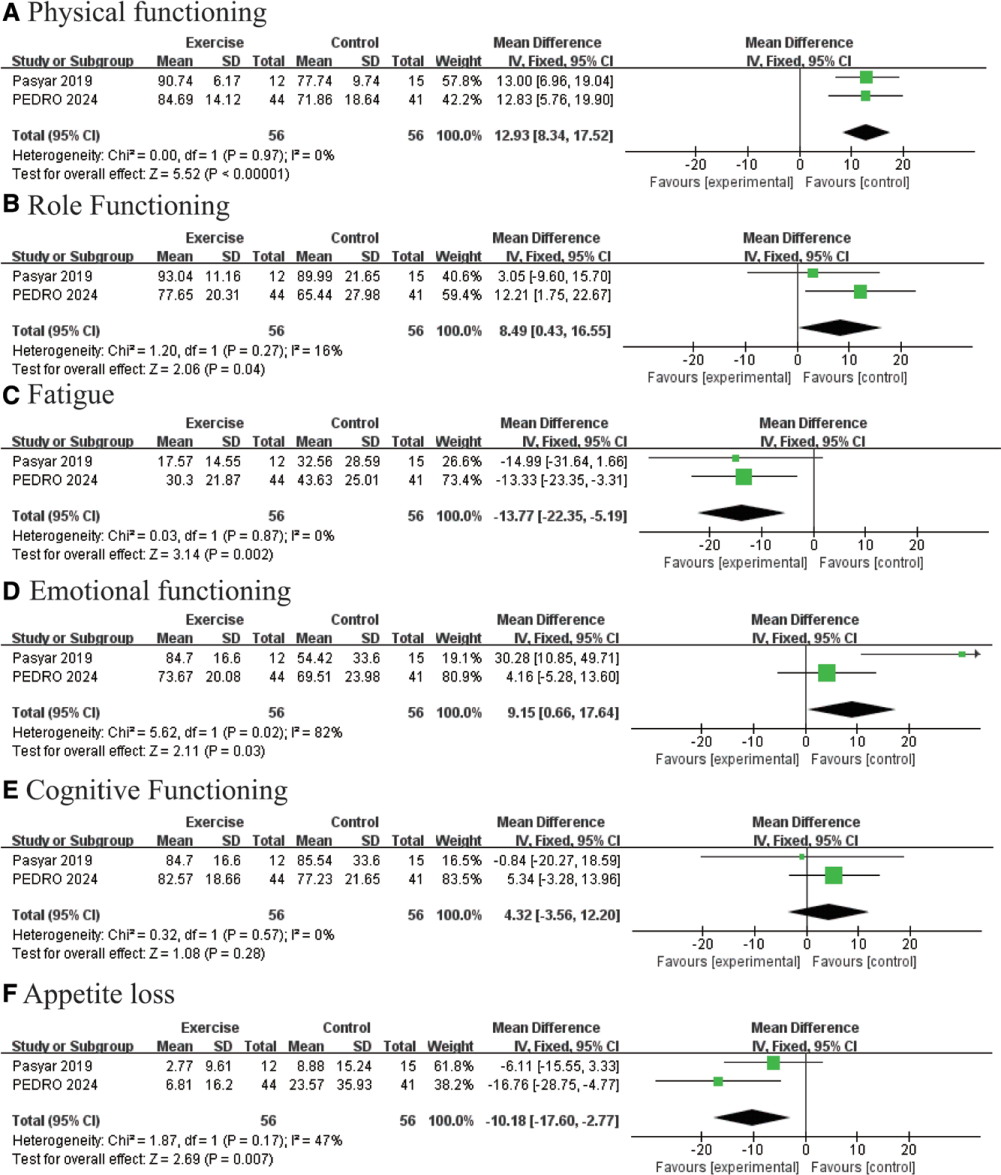
Effect of exercise on quality of life (QLQ-C30): (A) physical functioning; (B) role functioning; (C) fatigue reduction; (D) emotional functioning; (E) cognitive functioning; and (F) appetite loss.

Exercise interventions were essential in improving physical function and QoL in postoperative BC patients. The exercise group demonstrated significant improvements in physical functioning (WMD: 12.93, 95% CI [17.52, 18.34], *P* < .00001; Fig. 6A) and role functioning (WMD: 8.49, 95% CI [0.43, 16.55], *P* = .04; Fig. 6B). Additionally, exercise significantly alleviated fatigue (WMD: −13.77, 95% CI [−22.35, −5.19], *P* = .002; Fig. 6C), improved emotional functioning (WMD: 9.15, 95% CI [7.64, 10.66], *P* = .03; Fig. 6D), and reduced appetite loss (WMD: −10.18, 95% CI [−17.60, −2.77], *P* = .007; Fig. 6E). Although cognitive functioning showed a positive trend, it did not reach statistical significance (WMD: 4.32, 95% CI [−1.56, 12.20], *P* = .16; Fig. 6F). Overall, exercise interventions substantially enhanced the physical, emotional, and overall well-being of patients.

### 3.7. Upper limb function (DASH score)

**Figure 7.**
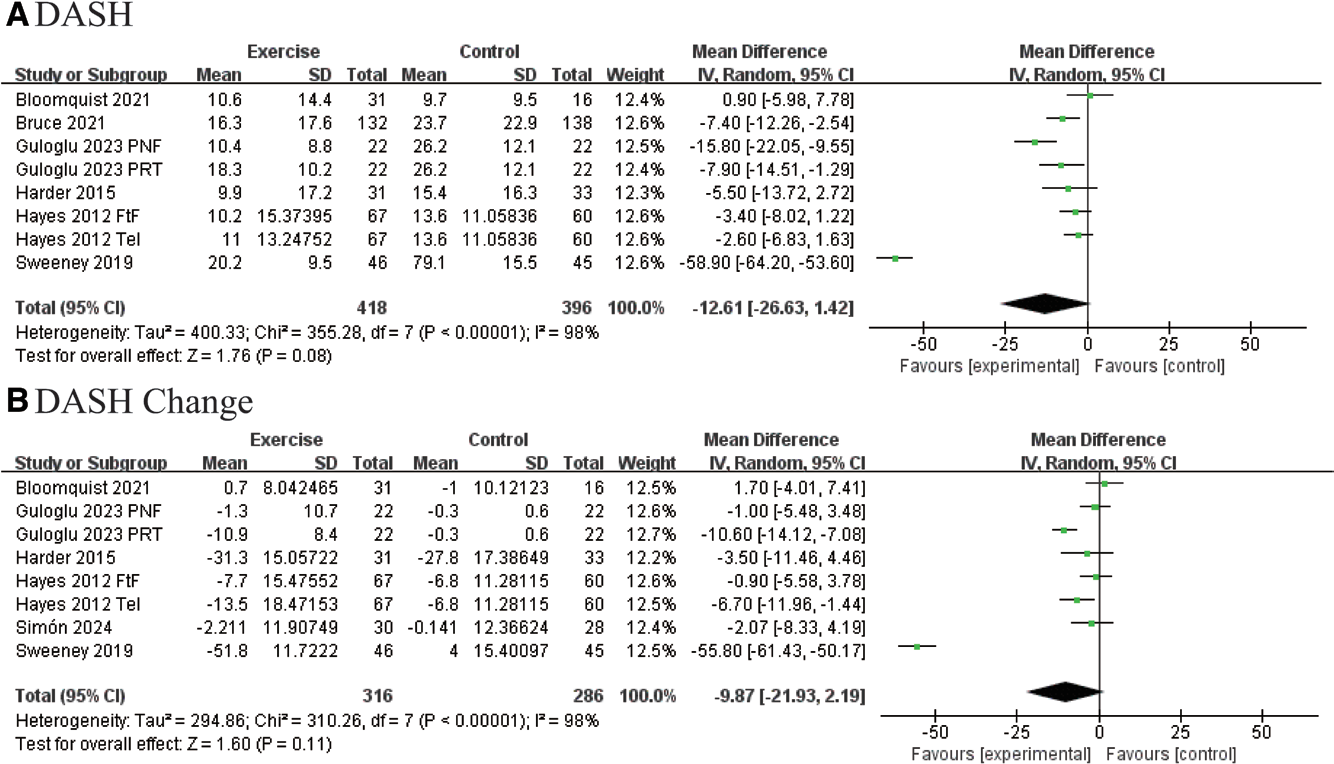
Effect of exercise on upper limb function (DASH score): (A) effect of exercise on DASH score at the end of the intervention; (B) change in DASH score from baseline.

This meta-analysis assessed the impact of exercise interventions on upper limb function in postoperative BC patients using the DASH scale. Results indicated a downward trend in DASH scores at the end of the intervention in the exercise group, suggesting some improvement in upper limb function compared to the control group. However, this difference was not statistically significant (*P* = .08, Fig. 7A). Similarly, while changes from baseline showed some improvement in the exercise group, the difference remained nonsignificant (*P* = .11, Fig. 7B). These findings suggest that although exercise may improve upper limb function recovery, the current evidence is not statistically conclusive, necessitating further high-quality research for validation.

## 4. Discussion

This systematic review integrated data from 22 RCTs encompassing a total of 2305 patients who underwent BC surgery. It systematically revealed the comprehensive benefits of exercise interventions in postoperative pain control, lymphedema management, upper limb functional recovery, and QoL improvement. The findings not only provide high-level evidence for existing clinical guidelines but also deepen the understanding of exercise rehabilitation theories from perspectives including pathophysiological mechanisms, rehabilitation timing, and individualized program design.

In terms of pain management, this study found that exercise interventions have a significant analgesic effect (MD = −0.49, *P* < .0001). The mechanisms may include: on the one hand, aerobic and resistance training can promote the release of β-endorphins, reduce levels of pro-inflammatory cytokines (such as IL-6 and TNF-α), and alleviate central sensitization responses^[37,38]^; on the other hand, exercise improves local blood circulation, facilitates the clearance of metabolic byproducts (such as lactic acid and bradykinin), and enhances tissue oxygenation, thus reducing the activity of acid-sensing ion channels and inhibiting the excitability of peripheral nociceptors.^[39–41]^ Furthermore, exercise may enhance parasympathetic tone and improve autonomic nervous system function, synergistically regulating the hypothalamic–pituitary–adrenal axis to form a multilayered analgesic regulatory network.^[42]^ Targeted shoulder joint exercises also demonstrated specific analgesic advantages (MD = −0.39, *P* = .04), suggesting that fascial release targeting the surgical area can reduce mechanical stimulation of nerve endings caused by scar adhesions, further alleviating local pain.

As for lymphedema management, the results showed that exercise interventions significantly reduced the risk of postoperative lymphedema (OR = 0.34, *P* = .0003), indicating a protective role of exercise on the lymphatic system after surgery. The mechanisms mainly include 2 aspects: firstly, exercise enhances the skeletal muscle pump, promoting the venous and lymphatic return of interstitial fluid and thus reducing local fluid retention. Periodic contraction of skeletal muscles during exercise increases interstitial pressure, assists venous valve closure, and facilitates lymphatic drainage, improving the local microcirculatory environment. Secondly, mechanical stimulation during exercise can increase the spontaneous contraction frequency and strength of lymphatic smooth muscle, further enhancing lymphatic return.^[43]^ Additionally, pathophysiological research suggests that exercise may modulate the vascular endothelial growth factor-C and its receptor VEGFR-3 signaling pathways, fostering lymphangiogenesis. It also inhibits the overproduction of pro-fibrotic factors, such as transforming growth factor-β1, which helps delay perilymphatic fibrosis and pathological remodeling, ultimately slowing the development of lymphedema.^[44]^ Upper arm circumference, a commonly used clinical assessment indicator, showed significant improvement in the exercise group (MD = −0.47, *P* = .02), further confirming the effectiveness of exercise in promoting venous-lymphatic return and alleviating local tissue fluid accumulation. However, improvements in extracellular fluid volume (*P* = .55) and L-Dex values obtained via bioimpedance analysis (*P* = .10) did not reach statistical significance. It is noteworthy that early implementation of exercise interventions post-surgery may achieve better outcomes. In the early postoperative phase, the lymphatic system has not yet undergone irreversible fibrosis and tissue remodeling and retains higher plasticity, making it more responsive to exercise-induced recovery and regeneration. Therefore, early intervention with scientifically designed exercise programs is crucial for postoperative recovery in BC patients.

Regarding upper limb functional recovery, this study further validated the significant benefits of exercise interventions. Patients in the exercise group showed greater improvements in shoulder muscle strength and grip strength compared to controls (grip strength MD = 2.35, *P* < .00001). This improvement not only reflects traditional physical adaptations such as enhanced joint flexibility and muscle strength restoration but also suggests deeper biological mechanisms. Previous studies have indicated that progressive load training can activate the mechanical target of rapamycin signaling pathway and promote the synthesis of myosin heavy chains, highlighting the positive effects of exercise rehabilitation at the molecular level.^[45]^ Shoulder joint ROM also improved to varying degrees in multiple directions, further supporting the role of exercise in promoting joint structure remodeling. Exercise may regulate the alignment of collagen fibers within the joint capsule, thereby alleviating tissue stiffness and joint contracture caused by postoperative immobilization. However, improvement in shoulder external rotation did not reach statistical significance (*P* = .06). Thus, future rehabilitation programs should consider incorporating neurodynamic techniques or targeted neural rehabilitation training to optimize the recovery of external rotation function. In addition, although the DASH score in the exercise group showed a downward trend, suggesting improvement in upper limb function, the difference was not statistically significant (*P* = .08). This may reflect the limited sensitivity of subjective assessment tools to detect minor functional changes or suggest that restoring functional independence after BC surgery requires prolonged intervention and consolidation training.

Following BC surgery, our meta-analysis confirmed that exercise interventions significantly enhance patients’ QoL. Based on the QLQ-C30 scale, patients in the exercise group showed significant improvements in physical functioning (MD = 12.93, *P* < .00001), role functioning (MD = 8.49, *P* = .04), and emotional functioning (MD = 9.15, *P* = .03), particularly in alleviating cancer-related fatigue (MD = −13.77, *P* = .002). Exercise also improved appetite (MD = −10.18, *P* = .007), which may be related to the modulation of the ghrelin-leptin signaling pathway, providing a new management approach for postoperative appetite loss. These results support the integration of exercise interventions into standardized postoperative rehabilitation systems for BC, especially in managing symptoms such as fatigue and appetite loss. Overall, exercise interventions play a vital role in the comprehensive rehabilitation of BC patients after surgery.

## 5. Conclusion

This systematic review and meta-analysis synthesizing current RCT evidence indicates that exercise interventions play an important and positive role in postoperative rehabilitation following BC surgery. Exercise can significantly alleviate postoperative pain, reduce the incidence of lymphedema, and effectively improve shoulder ROM and upper limb function, while also significantly enhancing patients’ overall QoL. Overall, exercise interventions demonstrate good safety and efficacy. Therefore, exercise should be considered an essential component of postoperative rehabilitation management for BC and should be incorporated into recovery plans as early as possible under professional guidance to optimize long-term functional recovery and QoL improvement. In future clinical practice, efforts should be made to further promote and standardize exercise rehabilitation interventions to advance the comprehensive recovery of patients after BC surgery.

## 6. Limitations

While summarizing the effects of exercise interventions after BC surgery, this study also has several limitations that require cautious interpretation. Firstly, there was considerable variation in the included interventions, such as the type of exercise (e.g., aerobic exercise, resistance training, flexibility exercises, shoulder-elbow specific exercises), frequency, duration, and intensity, leading to high heterogeneity in the overall intervention protocols, which may affect the pooled effect estimates for each outcome. Secondly, inconsistencies in evaluation tools and measurement time points across studies – such as different tools used for pain assessment (Visual Analog Scale, Numerical Rating Scale score, McGill Pain Questionnaire) and lymphedema assessment methods (volume measurement, circumference measurement, bioimpedance analysis) – increased the complexity of comparing results and may have introduced measurement bias. Moreover, some included studies had small sample sizes and short intervention or follow-up durations, leading to insufficient evidence regarding the long-term effects (such as the sustainability of functional recovery and the stability of QoL improvements). Small sample size studies may also exaggerate the intervention effects and pose a risk of reporting bias. Additionally, methodological quality issues in some studies – such as unclear descriptions of randomization, allocation concealment, and blinding – may have affected the overall evidence quality. Differences in patients’ baseline characteristics (e.g., type of surgery, whether they received chemo/radiotherapy, initial functional status) and the lack of adequate adjustment for these potential confounding factors in some studies may have interfered with intervention effects. Furthermore, due to language limitations, only English and some Chinese literature were included, potentially resulting in publication bias. Finally, the lack of standardization in intervention measures and outcome indicators prevented subgroup analyses exploring the moderating effects of exercise type, intensity, and patient characteristics, limiting the detailed interpretation and clinical applicability of the results. Therefore, future research should focus on improving the design of intervention protocols, unifying evaluation systems, and standardizing study designs to provide higher-quality evidence for exercise rehabilitation after BC surgery.

## Author contributions

**Conceptualization:** Tingyu Xue, Li Zhang, Dairong Zhang.

**Data curation:** Tingyu Xue.

**Formal analysis:** Tingyu Xue, Li Zhang.

**Funding acquisition:** Li Zhang.

**Investigation:** Li Zhang.

**Methodology:** Li Zhang.

**Writing – original draft:** Tingyu Xue, Dairong Zhang.

**Writing – review & editing:** Tingyu Xue, Dairong Zhang.

## Supplementary Material



## References

[1] Barzaman K Karami J Zarei Z. Breast cancer: biology, biomarkers, and treatments. Int Immunopharmacol. 2020;84:106535. 32361569 10.1016/j.intimp.2020.106535

[2] Park J Rodriguez JL O’Brien KM. Health-related quality of life outcomes among breast cancer survivors. Cancer. 2021;127:1114–25. 33237602 10.1002/cncr.33348 PMC8035208

[3] Al-Hilli Z Wilkerson A. Breast surgery: management of postoperative complications following operations for breast cancer. Surg Clin North Am. 2021;101:845–63. 34537147 10.1016/j.suc.2021.06.014

[4] Rockson SG. Lymphedema after breast cancer treatment. N Engl J Med. 2018;379:1937–44. 30428297 10.1056/NEJMcp1803290

[5] Kline-Quiroz C Nori P Stubblefield MD. Cancer rehabilitation: acute and chronic issues, nerve injury, radiation sequelae, surgical and chemo-related, part 1. Med Clin North Am. 2020;104:239–50. 32035566 10.1016/j.mcna.2019.10.004

[6] Min J Kim JY Ryu J. Early implementation of exercise to facilitate recovery after breast cancer surgery: a randomized clinical trial. JAMA Surg. 2024;159:872–80. 38837150 10.1001/jamasurg.2024.1633 PMC11154354

[7] Ye X-X Ren Z-Y Vafaei S. Effectiveness of baduanjin exercise on quality of life and psychological health in postoperative patients with breast cancer: a systematic review and meta-analysis. Integr Cancer Ther. 2022;21:15347354221104092. 35699146 10.1177/15347354221104092 PMC9202258

[8] Dams L Van der Gucht E Devoogdt N. Effect of pain neuroscience education after breast cancer surgery on pain, physical, and emotional functioning: a double-blinded randomized controlled trial (EduCan trial). Pain. 2023;164:1489–501. 36637138 10.1097/j.pain.0000000000002838

[9] Wilson DJ. Exercise for the patient after breast cancer surgery. Semin Oncol Nurs. 2017;33:98–105. 28063632 10.1016/j.soncn.2016.11.010

[10] McNeely ML Campbell K Ospina M. Exercise interventions for upper-limb dysfunction due to breast cancer treatment. Cochrane Database Syst Rev. 2010:CD005211. 20556760 10.1002/14651858.CD005211.pub2 PMC12861582

[11] De Groef A Van Kampen M Dieltjens E. Effectiveness of postoperative physical therapy for upper-limb impairments after breast cancer treatment: a systematic review. Arch Phys Med Rehabil. 2015;96:1140–53. 25595999 10.1016/j.apmr.2015.01.006

[12] Antunes P Joaquim A Sampaio F. Exercise training benefits health-related quality of life and functional capacity during breast cancer chemotherapy: a randomized controlled trial. Med Sci Sports Exerc. 2024;56:600–11. 38051110 10.1249/MSS.0000000000003341

[13] Luo D Wan X Liu J Tong T. Optimally estimating the sample mean from the sample size, median, mid-range, and/or mid-quartile range. Stat Methods Med Res. 2018;27:1785–805. 27683581 10.1177/0962280216669183

[14] Wan X Wang W Liu J Tong T. Estimating the sample mean and standard deviation from the sample size, median, range and/or interquartile range. BMC Med Res Methodol. 2014;14:135. 25524443 10.1186/1471-2288-14-135 PMC4383202

[15] Ammitzbøll G Andersen KG Bidstrup PE. Effect of progressive resistance training on persistent pain after axillary dissection in breast cancer: a randomized controlled trial. Breast Cancer Res Treat. 2020;179:173–83. 31605312 10.1007/s10549-019-05461-z

[16] Bruce J Mazuquin B Canaway A. Exercise versus usual care after non-reconstructive breast cancer surgery (UK PROSPER): multicentre randomised controlled trial and economic evaluation. BMJ. 2021;375:e066542. 34759002 10.1136/bmj-2021-066542 PMC8579424

[17] Guloglu S Basim P Algun ZC. Efficacy of proprioceptive neuromuscular facilitation in improving shoulder biomechanical parameters, functionality, and pain after axillary lymph node dissection for breast cancer: a randomized controlled study. Complement Ther Clin Pract. 2023;50:101692. 36528984 10.1016/j.ctcp.2022.101692

[18] Huo M Zhang X Fan J. Short-term effects of a new resistance exercise approach on physical function during chemotherapy after radical breast cancer surgery: a randomized controlled trial. BMC Womens Health. 2024;24:160. 38443932 10.1186/s12905-024-02989-1 PMC10913245

[19] Hayes SC Rye S Disipio T. Exercise for health: a randomized, controlled trial evaluating the impact of a pragmatic, translational exercise intervention on the quality of life, function and treatment-related side effects following breast cancer. Breast Cancer Res Treat. 2013;137:175–86. 23139058 10.1007/s10549-012-2331-y

[20] Harder H Langridge C Solis-Trapala I. Post-operative exercises after breast cancer surgery: results of a RCT evaluating standard care versus standard care plus additional yoga exercise. Eur J Integr Med. 2015;7:202–10.

[21] Bloomquist K Krustrup P Fristrup B. Effects of football fitness training on lymphedema and upper-extremity function in women after treatment for breast cancer: a randomized trial. Acta Oncol. 2021;60:392–400. 33423594 10.1080/0284186X.2020.1868570

[22] Uth J Fristrup B Sørensen V. One year of football fitness improves L1-L4 BMD, postural balance, and muscle strength in women treated for breast cancer. Scand J Med Sci Sports. 2021;31:1545–57. 33794005 10.1111/sms.13963

[23] Kilbreath SL Refshauge KM Beith JM. Upper limb progressive resistance training and stretching exercises following surgery for early breast cancer: a randomized controlled trial. Breast Cancer Res Treat. 2012;133:667–76. 22286332 10.1007/s10549-012-1964-1

[24] Kilbreath SL Ward LC Davis GM. Reduction of breast lymphoedema secondary to breast cancer: a randomised controlled exercise trial. Breast Cancer Res Treat. 2020;184:459–67. 32812177 10.1007/s10549-020-05863-4

[25] Loudon A Barnett T Piller N Immink MA Williams AD. Yoga management of breast cancer-related lymphoedema: a randomised controlled pilot-trial. BMC Complement Altern Med. 2014;14:214. 24980836 10.1186/1472-6882-14-214 PMC4083036

[26] Majed M Neimi CA Youssef SM Takey KA Badr LK. The impact of therapeutic exercises on the quality of life and shoulder range of motion in women after a mastectomy, an RCT. J Cancer Educ. 2022;37:843–51. 33219500 10.1007/s13187-020-01894-z

[27] Pasyar N Barshan Tashnizi N Mansouri P Tahmasebi S. Effect of yoga exercise on the quality of life and upper extremity volume among women with breast cancer related lymphedema: a pilot study. Eur J Oncol Nurs. 2019;42:103–9. 31479846 10.1016/j.ejon.2019.08.008

[28] Park J-H. The effects of complex exercise on shoulder range of motion and pain for women with breast cancer-related lymphedema: a single-blind, randomized controlled trial. Breast Cancer. 2017;24:608–14. 28008557 10.1007/s12282-016-0747-7

[29] Esteban-Simón A Díez-Fernández DM Rodríguez-Pérez MA Artés-Rodríguez E Casimiro-Andújar AJ Soriano-Maldonado A. Does a resistance training program affect between-arms volume difference and shoulder-arm disabilities in female breast cancer survivors? The role of surgery type and treatments. Secondary outcomes of the EFICAN trial. Arch Phys Med Rehabil. 2024;105:647–54. 38043674 10.1016/j.apmr.2023.11.010

[30] Sweeney FC Demark-Wahnefried W Courneya KS. Aerobic and resistance exercise improves shoulder function in women who are overweight or obese and have breast cancer: a randomized controlled trial. Phys Ther. 2019;99:1334–45. 31309977 10.1093/ptj/pzz096 PMC6821226

[31] Tambour M Holt M Speyer A Christensen R Gram B. Manual lymphatic drainage adds no further volume reduction to complete decongestive therapy on breast cancer-related lymphoedema: a multicentre, randomised, single-blind trial. Br J Cancer. 2018;119:1215–22. 30353049 10.1038/s41416-018-0306-4 PMC6251025

[32] Todd J Scally A Dodwell D Horgan K Topping A. A randomised controlled trial of two programmes of shoulder exercise following axillary node dissection for invasive breast cancer. Physiotherapy. 2008;94:265–73.

[33] Williams AF Vadgama A Franks PJ, . A randomized controlled crossover study of manual lymphatic drainage therapy in women with breast cancer-related lymphoedema. Eur J Cancer Care. 2003;11:254–61. 10.1046/j.1365-2354.2002.00312.x 12492462

[34] Torres Lacomba M Yuste Sanchez MJ Zapico Goni A, . Effectiveness of early physiotherapy to prevent lymphoedema after surgery for breast cancer: randomised, single blinded, clinical trial. BMJ. 2010;340:b5396. 20068255 10.1136/bmj.b5396 PMC2806631

[35] Bloomquist K Krustrup P Fristrup B, . Effects of football fitness training on lymphedema and upper-extremity function in women after treatment for breast cancer: a randomized trial. Acta Oncologica. 2021;60:392–400. 33423594 10.1080/0284186X.2020.1868570

[36] Antunes P Joaquim A Sampaio F, . Exercise training benefits health-related quality of life and functional capacity during breast cancer chemotherapy: a randomized controlled trial. Med Sci Sports Exerc. 2023;56:600–11. 38051110 10.1249/MSS.0000000000003341

[37] Hughes L Patterson SD. The effect of blood flow restriction exercise on exercise-induced hypoalgesia and endogenous opioid and endocannabinoid mechanisms of pain modulation. J Appl Physiol (1985). 2020;128:914–24. 32105522 10.1152/japplphysiol.00768.2019

[38] Sharif K Watad A Bragazzi NL Lichtbroun M Amital H Shoenfeld Y. Physical activity and autoimmune diseases: get moving and manage the disease. Autoimmun Rev. 2018;17:53–72. 29108826 10.1016/j.autrev.2017.11.010

[39] Khataei T Harding AMS Janahmadi M. ASICs are required for immediate exercise-induced muscle pain and are downregulated in sensory neurons by exercise training. J Appl Physiol (1985). 2020;129:17–26. 32463731 10.1152/japplphysiol.00033.2020

[40] Powers SK Quindry JC Kavazis AN. Exercise-induced cardioprotection against myocardial ischemia–reperfusion injury. Free Radic Biol Med. 2008;44:193–201. 18191755 10.1016/j.freeradbiomed.2007.02.006

[41] Hoppeler H. Molecular networks in skeletal muscle plasticity. J Exp Biol. 2016;219:205–13. 26792332 10.1242/jeb.128207

[42] Ozhanli Y Akyuz N. The effect of progressive relaxation exercise on physiological parameters, pain and anxiety levels of patients undergoing colorectal cancer surgery: a randomized controlled study. J Perianesth Nurs. 2022;37:238–46. 34903440 10.1016/j.jopan.2021.08.008

[43] Chang CJ Cormier JN. Lymphedema interventions: exercise, surgery, and compression devices. Semin Oncol Nurs. 2013;29:28–40. 23375064 10.1016/j.soncn.2012.11.005

[44] Park HS Song Y Lee J-H Oh K-R Park H Kang H. The role of exercise in promoting lymphangiogenesis and extracellular matrix synthesis in lymphedema-induced tissue injury. Mol Biol Rep. 2024;52:50. 39676093 10.1007/s11033-024-10149-9

[45] Yasuda T. Selected methods of resistance training for prevention and treatment of sarcopenia. Cells. 2022;11:1389. 35563694 10.3390/cells11091389 PMC9102413

