# Supplementary material for: Exercise-based interventions for postoperative rehabilitation in breast cancer patients: A systematic review and meta-analysis of randomized controlled trials
Source: Medicine (Baltimore). 2025 Aug 22;104(34):e43705. doi: 10.1097/MD.0000000000043705 (PMC12384885; doi:10.1097/MD.0000000000043705)

Figure S1. Effect of Exercise on Range of Motion (ROM) Change: (A) ROM Flexion; (B) ROM Extension; (C) ROM Abduction; (D) ROM External rotation; (E) ROM Internal rotation.

A ROM Flexion

Study or Subgroup	Exercise Mean	Exercise SD	Exercise Total	Control Mean	Control SD	Control Total	Weight	Mean Difference IV, Random, 95% CI
Huo 2024 Conventional	146.3	20.7	50	132.4	28.1	61	15.4%	13.90 [4.81, 22.99]
Huo 2024 NJF	149	22.6	51	132.4	28.1	61	15.3%	16.60 [7.21, 25.99]
Majed 2020	167.97	4.09	30	159.92	1.73	30	18.0%	8.05 [6.46, 9.64]
Min 2024	172.3	12.2	28	166.5	14.4	28	16.4%	5.80 [-1.19, 12.79]
Park 2017	160.66	3.96	32	157.16	5.54	31	17.8%	3.50 [1.12, 5.88]
Sweeney 2019	172.1	14	50	129.3	13.2	50	17.1%	42.80 [37.47, 48.13]
**Total (95% CI)**			**241**			**261**	**100.0%**	**15.01 [5.62, 24.39]**

Heterogeneity: Tau² = 127.07; Chi² = 180.50, df = 5 (P < 0.00001); I² = 97%
Test for overall effect: Z = 3.13 (P = 0.002)

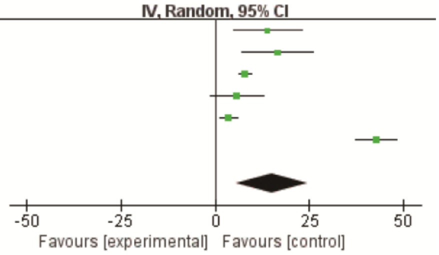

B ROM Extension

Study or Subgroup	Exercise Mean	Exercise SD	Exercise Total	Control Mean	Control SD	Control Total	Weight	Mean Difference IV, Random, 95% CI
Huo 2024 Conventional	46.3	6.8	50	44.6	7	61	19.6%	1.70 [-0.88, 4.28]
Huo 2024 NJF	46.4	5.2	51	44.6	7	61	20.0%	1.80 [-0.46, 4.06]
Majed 2020	53.07	2.12	30	49.03	1.25	30	21.3%	4.04 [3.16, 4.92]
Min 2024	48.4	7.6	28	34.4	7	28	17.7%	14.00 [10.17, 17.83]
Park 2017	36.5	1.95	32	26.74	1.55	31	21.3%	9.76 [8.89, 10.63]
**Total (95% CI)**			**191**			**211**	**100.0%**	**6.11 [2.33, 9.90]**

Heterogeneity: Tau² = 17.29; Chi² = 128.76, df = 4 (P < 0.00001); I² = 97%
Test for overall effect: Z = 3.17 (P = 0.002)

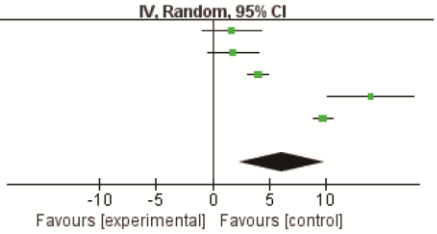

C ROM Abduction

Study or Subgroup	Exercise Mean	Exercise SD	Exercise Total	Control Mean	Control SD	Control Total	Weight	Mean Difference IV, Fixed, 95% CI
Huo 2024 Conventional	42.5	3.8	50	39.8	6.6	61	29.4%	2.70 [0.74, 4.66]
Huo 2024 NJF	41.4	4.4	51	39.8	6.6	61	27.0%	1.60 [-0.45, 3.65]
Park 2017	37.69	1.99	32	35.19	4.14	31	43.6%	2.50 [0.89, 4.11]
**Total (95% CI)**			**133**			**153**	**100.0%**	**2.32 [1.25, 3.38]**

Heterogeneity: Chi² = 0.67, df = 2 (P = 0.72); I² = 0%
Test for overall effect: Z = 4.26 (P < 0.0001)

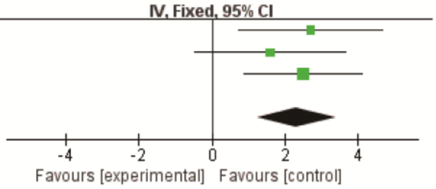

D ROM External Rotation

Study or Subgroup	Exercise Mean	Exercise SD	Exercise Total	Control Mean	Control SD	Control Total	Weight	Mean Difference IV, Random, 95% CI
Huo 2024 Conventional	54.3	7.1	50	52.7	8.4	61	25.0%	1.60 [-1.28, 4.48]
Huo 2024 NJF	55.9	8	51	52.7	8.4	61	25.0%	3.20 [0.16, 6.24]
Park 2017	58.53	7.18	32	46.87	3.12	31	25.1%	11.66 [8.94, 14.38]
Sweeney 2019	78.5	8.5	50	52.1	7.8	50	24.9%	26.40 [23.20, 29.60]
**Total (95% CI)**			**183**			**203**	**100.0%**	**10.70 [0.12, 21.29]**

Heterogeneity: Tau² = 114.33; Chi² = 154.18, df = 3 (P < 0.00001); I² = 98%
Test for overall effect: Z = 1.98 (P = 0.05)

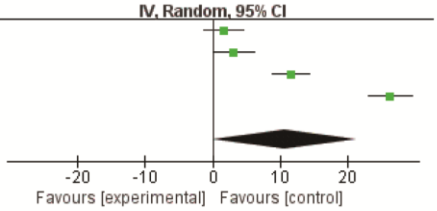

E ROM Internal Rotation

Study or Subgroup	Exercise Mean	Exercise SD	Exercise Total	Control Mean	Control SD	Control Total	Weight	Mean Difference IV, Random, 95% CI
Huo 2024 Conventional	59.7	9.8	50	62.5	9.5	61	31.6%	-2.80 [-6.41, 0.81]
Huo 2024 NJF	60.1	9.4	51	62.5	9.5	61	31.9%	-2.40 [-5.91, 1.11]
Park 2017	31.63	4.73	32	27.84	1.95	31	36.5%	3.79 [2.01, 5.57]
**Total (95% CI)**			**133**			**153**	**100.0%**	**-0.27 [-5.12, 4.58]**

Heterogeneity: Tau² = 15.95; Chi² = 16.50, df = 2 (P = 0.0003); I² = 88%
Test for overall effect: Z = 0.11 (P = 0.91)

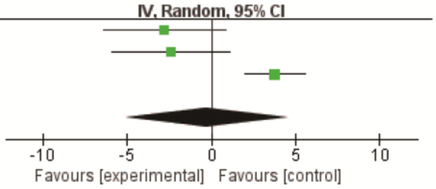

Supplement: Supplementary file 2 [file medi-104-e43705-s002.pdf]
